# Accuracy of holmium-166 SPECT/CT quantification over a large range of activities

**DOI:** 10.1186/s40658-024-00683-7

**Published:** 2024-09-26

**Authors:** Lovisa E. L. Westlund Gotby, Daphne Lobeek, Joey Roosen, Maarten de Bakker, Mark W. Konijnenberg, J. Frank W. Nijsen

**Affiliations:** 1 Department of Medical Imaging, Radboud University Medical Center, Nijmegen, The Netherlands; 2 Department of Radiology and Nuclear Medicine, Erasmus University Medical Center, Rotterdam, The Netherlands

**Keywords:** SPECT quantification, Holmium-166, Conversion factor, Absolute quantification, Recovered activity

## Abstract

**Background:**

Quantitative imaging is a crucial step for dosimetry in radionuclide therapies. Traditionally, SPECT/CT imaging is quantified based on scanner-specific conversion factors or self-calibration, but recently absolute quantification methods have been introduced in commercial SPECT reconstruction software (Broad Quantification, Siemens Healthineers). In this phantom study we investigate the accuracy of three quantification methods for holmium-166 SPECT/CT imaging, and provide recommendations for clinical dosimetry.

**Methods:**

One cylindrical phantom, filled with a homogeneous holmium-166-chloride activity concentration solution, was imaged at one time point to determine a scanner-specific conversion factor, and to characterize the spatial dependency of the activity concentration recovery. One Jaszczak phantom with six fillable spheres, 10:1 sphere-to-background ratio, was imaged over a large range of holmium-166 activities (61-3130 MBq). The images were reconstructed with either an ordered subset expectation maximization (OSEM, Flash3D-reconstruction; scanner-specific quantification or self-calibration quantification) or an ordered subset conjugate gradient (OSCG, xSPECT-reconstruction; Broad Quantification) algorithm. These three quantification methods were compared for the data of the Jaszczak phantom and evaluated based on whole phantom recovered activity, activity concentration recovery coefficients (ACRC), and recovery curves.

**Results:**

The activity recovery in the Jaszczak phantom was 28–115% for the scanner-specific, and 57–97% for the Broad Quantification quantification methods, respectively. The self-calibration-based activity recovery is inherently always 100%. The ACRC for the largest sphere (Ø60 mm, ~ 113 mL) ranged over (depending on the activity level) 0.22–0.89, 0.76–0.86, 0.39–0.72 for scanner-specific, self-calibration and Broad Quantification, respectively.

**Conclusion:**

Of the three investigated quantification methods, the self-calibration technique produces quantitative SPECT images with the highest accuracy in the investigated holmium-166 activity range.

**Supplementary Information:**

The online version contains supplementary material available at 10.1186/s40658-024-00683-7.

## Background

Over the past decades, the medical isotope holmium-166 (^166^Ho) has become a widely explored therapeutic radionuclide [1]. Its primary clinical application is the ^166^Ho poly-L-lactic acid microsphere used in medical procedures such as transarterial radioembolization (TARE) or direct intratumoral injection of the microspheres [2]. During TARE, radioactive microspheres are injected in (a subbranch of) the hepatic artery, and are distributed through the perfused liver volume via the blood flow. The microspheres ultimately lodge in the arterioles, where they locally irradiate the tissue [3].

^166^Ho emits high-energy beta particles (T_1/2_: 26.8 h; max energy 1.77 MeV/50.5%, 1.85 MeV/48.2%),a primary gamma peak at 80.57 keV/6.55%, which is typically used for planar scintigraphy and single photon emission computed tomography with computed tomography (SPECT/CT) imaging, and a spectrum of high energy gammas with lower abundance (1.38 MeV/0.93%, 1.58 MeV/0.19%, 1.66 MeV/0.12%) [4, 5]. Scattered high energy photons, bremsstrahlung photons from the beta radiation, as well as characteristic x-rays from the lead collimator [6], all contribute towards contamination of the main ^166^Ho photopeak window. The scattered high energy, and the bremsstrahlung photons can be estimated for using window-based scatter correction in the reconstruction of the SPECT images, but since the spectrum of the characteristic lead x-rays overlaps with both the main ^166^Ho photopeak window as well as possible adjacent scatter windows, these interactions can only be accounted for using a dedicated Monte Carlo-based scatter correction method. In ^166^Ho-TARE, SPECT imaging is used for both pre-treatment dosimetry and therapy planning [7], as well as for post-treatment dosimetry and dose-response evaluation [8]. As the ^166^Ho microspheres are paramagnetic, magnetic resonance imaging-based (MRI) dosimetry can also be performed, as an alternative to traditional nuclear medicine based dose evaluation [9, 10]. For treatment optimization based on patient dosimetry, a thorough understanding of dose-response relationships after radionuclide therapy, and verification of the absorbed doses, is vital [11]. This requires standardized and optimized quantitative imaging and dosimetry protocols, for clinical studies to incorporate [8].

Although SPECT/CT-based dosimetry for ^166^Ho-TARE treatment planning and evaluation is considered the current gold standard [6], its value is limited by the low spatial resolution, motion artifacts, and the lack of standardized quantification. Traditionally, SPECT/CT images have been quantified based on a pre-determined calibration factor measured in a phantom [12–16]. One major drawback of this method is that it is phantom dependent and/or scanner-specific which can make it cumbersome to quantitively compare absorbed doses in multi-scanner, multi-center studies. An alternative method is to quantify the SPECT/CT images based on a patient self-calibration conversion factor [4, 15]. This method is uniquely applicable for localized radionuclide therapies such as TARE, as all the activity can be imaged within one field-of-view of the SPECT (possible extrahepatic deposition can be estimated for based on SPECT or planar scintigraphy) and because the microspheres have no biological half-life (permanent entrapment of the radioactive particles). A single bed position, single time point SPECT/CT scan is therefore sufficient for the determination of the self-calibration conversion factor. A third option for quantification, recently introduced by some vendors, is absolute SPECT quantification in commercial software [17, 18], which could potentially aid in increasing the reproducibility and the reliability of the reported absorbed doses.

In this phantom study, we quantitively compare three different ^166^Ho-SPECT/CT-based quantification methods; scanner-specific conversion, self-calibration, and a commercial software for absolute quantification (Broad Quantification, Siemens Healthineers, Erlangen, Germany). Furthermore, we evaluate the accuracy of these quantification methods for the purpose of post-treatment dosimetry over a large range of ^166^Ho activities, and we provide recommendations for post-treatment absorbed dose evaluation in clinical practice.

**Table 1:** Activity in the phantoms present at injection and at the time point of scanning. The dead-time [%] was read from the workstation during the data acquisition. Measurements at time points marked with * were repeated three times

Phantom	Time point	Δt [h]	Activity in phantom [MBq]	Activity concentration in phantom [MBq/mL]		Dead-time as indicated by the scanner [%]
Cylindrical homogeneous phantom	Injection	0	311	0.049		-
	1	8.5	250	0.040		~ 2.25
Jaszczak phantom	Injection	0	3260	Spheres	Background	-
				4.10	0.41	
	1	1.5	3130	3.94	0.39	34–45
	2	5.1	2853	3.59	0.36	28–42
	3	10.2	2503	3.15	0.31	25–37
	4	14.6	2232	2.81	0.28	22–36
	5	24.4	1736	2.18	0.22	18–29
	6	28.6	1554	1.96	0.20	20–27
	7	33.5	1370	1.72	0.17	19–25
	8	37.7	1230	1.55	0.15	16–23
	9	48.2	938	1.18	0.12	13–18
	10*	58.0, 59.2, 60.3	727, 706, 687	0.91, 0.89, 0.86	0.091, 0.089, 0.086	10–15
	11	75.4	464	0.58	0.058	7–10
	12*	81.9, 83.0, 84.1	393, 382, 371	0.49, 0.48, 0.47	0.049, 0.048, 0.047	5–8
	13*	104.8, 105.9, 106.9	217, 211, 206	0.27, 0.27, 0.26	0.027, 0.027, 0.026	3–5
	14*	151.9, 153.1, 154.2	64, 62, 61	0.081, 0.078, 0.076	0.0081, 0.0078, 0.0076	0.5–2

## Methods

### Phantom preparation

**Fig. 1:**
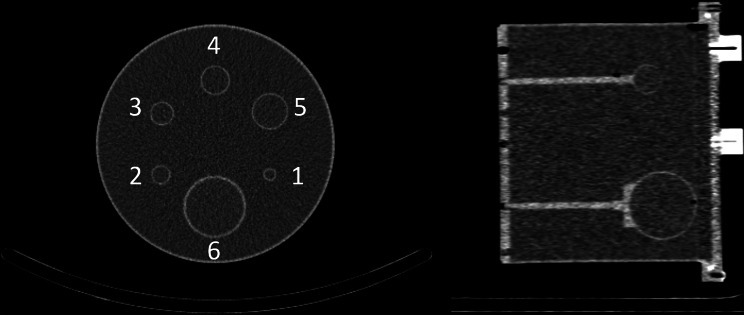
CT images of the Jaszczak phantom with the fillable spheres. Axial view on the left and sagittal view on the right. The sphere numbering presented in this image is used throughout this paper

Two phantoms were utilized in this study. Firstly, one cylindrical phantom was filled with a homogeneous distribution of ^166^Ho-chloride dissolved in demineralized water (HolmiumSolution, Quirem Medical B.V., Deventer, The Netherlands). This phantom had an inner diameter of 20 cm, an inner height of 20 cm, a volume of 6283 mL, and an activity concentration of 0.049 MBq/mL at the injection time point. The second phantom was a flanged, rod-less, Jaszczak phantom with six fillable spheres, see Fig. 1 for sphere numbering and configuration. The background compartment of the Jaszczak phantom had a volume of approximately 6.7 L and the fillable spheres had inner diameters of 9.9, 15.4, 19.8, 24.8, 31.3, 60 mm (and approximate volumes 0.5, 2.0, 4.0, 8.0, 16.0, 113 mL). The Jaszczak phantom was also filled with homogeneous solutions of ^166^Ho-chloride. The activity concentration was 4.10 MBq/mL in the spheres and 0.41 MBq/mL in the background compartment at the injection time point, resulting in a sphere-to-background activity concentration ratio ($\:\text{S}:\text{B}{\text{G}}_{\text{r}\text{a}\text{t}\text{i}\text{o}}$) of 10:1. The total activity in the Jaszczak phantom ranged from 3.26 GBq to 61 MBq throughout the experiment, see Table 1. The ^166^Ho activities were measured in a dose calibrator (manufactured in 2019, equipped with ionization chamber VIK-202, Comecer, Joure, The Netherlands). In both phantoms the ^166^Ho-chloride solution was saturated with 50 mM ethylenediaminetetraacetic acid to bind the holmium ions, forming water-soluble complexes.

### SPECT/CT system

Both phantoms were imaged using a Symbia Intevo Bold SPECT/CT scanner equipped with the proprietary absolute quantification software Broad Quantification and the additional TrueCalc software (Siemens Healthineers, Erlangen, Germany). The purpose of TrueCalc is to improve the detector response at high count rates by reducing the impact of detector dead-time and give consistent quantitative accuracy across a wide range of activities [17, 19, 20]. The SPECT/CT scanner was manufactured and installed in 2019, uses software version VB22A, features a dual-headed detector configuration with a field-of-view of 53.3$\:\times\:$38.7 cm^2^ and a NaI crystal with a thickness of 9.5 mm. Prior to the measurements the SPECT/CT scanner had been peaked with a ~ 5 MBq ^166^Ho point source, and the Broad Quantification calibration procedure for ^166^Ho had been carried out in accordance with the operator manual, see supplementary information.

### Data acquisition

Imaging of the homogeneous phantom was acquired at one time point (total activity of 250 MBq at scanning). Imaging of the Jaszczak phantom was acquired at 14 different time points with three repetitions at the 10th, 12th, 13th, and 14th time point. The dead-time [%] estimate was read from the workstation during the acquisition of the data (‘Tomo Acquisition’-activity, ‘Analyzer’-tab), see Table 1. All SPECT data were acquired with the following settings: a photopeak energy window at 81 keV (15% width), two adjacent scatter windows (8% width), non-circular orbit over 360°, step and shoot, 2 × 60 projections, 20 s projection time, and a 128 × 128 matrix size. Parallel hole, Medium Energy Low Penetration (MELP) collimators were used, and the checkbox for ‘Enable Quantitative Acquisition’ had been ticked. When acquiring quantitative data, with the additional dead-time correction software (TrueCalc), the SPECT scanner generates two different projection datasets per SPECT acquisition; one dataset that is corrected for the estimated count losses due to dead-time effects, and a second dataset that is uncorrected. The measured counting rate performance per time point, for these two projection datasets, can be found in the Supplemental information. The dead-time estimates, presented in Table 1, are unaffected by the TrueCalc software. Additionally, low-dose CT data for attenuation correction and structural information were acquired for each dataset. The positioning of the Jaszczak phantom was standardized across all measurements, see Fig. 1.

### Image reconstruction

The acquired SPECT data for both phantoms were reconstructed using two different Siemens proprietary algorithms; Flash3D and xSPECT. The widely used Flash3D method is a 3D ordered subset expectation maximization (OSEM) method (utilized the uncorrected projection data). For Flash3D, the data were reconstructed with 10 iterations 8 subsets, and a voxel size of $\:4.80\times\:4.80\times\:4.80\:\text{m}{\text{m}}^{\text{3}}$. The xSPECT algorithm utilizes an ordered subset conjugate gradient (OSCG) method and Broad Quantification was enabled, meaning that the images were reconstructed in the unit of Bq/mL. Additionally, the xSPECT algorithm also applied the TrueCalc count loss correction in the image reconstruction (utilized the projection data corrected for count losses). For xSPECT, the data were reconstructed with 36 iterations 1 subset, a voxel size of $\:4.88\times\:4.88\times\:4.88\:\text{m}{\text{m}}^{\text{3}}$, and decay corrected back to the injection time point. Triple energy window scatter correction (automatic scatter window weight of 0.94) and CT based attenuation correction was applied for both reconstruction methods. If nothing else is indicated, no post-reconstruction filtering was applied to the Flash3D data and a 15 mm full width at half maximum (FWHM) post-reconstruction filtering was applied to the xSPECT data. See supplementary information for the choice of image reconstruction parameters.

### Data analysis

All image delineation and all data analysis were performed using MATLAB (R2021a, The MathWorks Inc., Natick, Massachusetts).

#### Image delineation

All volumes-of-interests (VOIs) for spheres, background and whole phantom volumes, were semi-automatically created based on the known phantom geometry and CT based positioning on a high resolution CT (voxel size $\:0.98\times\:0.98\times\:1.5\:\text{m}{\text{m}}^{\text{3}}$). The background VOI in the Jaszczak phantom was defined by subtracting the sphere VOIs from the whole phantom VOI. To minimize loss of data precision in the analysis, the reconstructed SPECT data were transformed to the high resolution CT voxel space by means of an affine transformation and nearest neighbor interpolation. All analyses were carried out using the high resolution grid.

#### SPECT quantification

Three methods for quantifying the SPECT data for the Jaszczak phantom were compared in this study;

1. Scanner-specific conversion factor measured in the Flash3D-reconstruction of the cylindrical homogeneous phantom (CF_homogeneous_) [12–16],
2. Self-calibration based on the Flash3D-reconstruction and delineation of the Jaszczak phantom for each dataset (CF_self_) [4, 15], and.
3. Broad Quantification absolute quantification software (based on xSPECT-reconstruction).

For the two Flash3D-based quantification methods, the conversion factor (CF) was calculated using the following formula;$\:\text{C}\text{F}=\frac{\left(\frac{{\mu\:}}{\text{t}\cdot\:\text{n}\cdot\:{\text{V}}_{\text{v}\text{o}\text{x}\text{e}\text{l}}}\right)}{\left(\frac{\text{A}}{{\text{V}}_{\text{V}\text{O}\text{I}}}\right)}\hspace{1em}\hspace{1em}\left[\frac{\text{cps}\text{/}\text{mL}}{\text{MBq}\text{/}\text{mL}}\right],\hspace{1em}$

where $\:{\mu\:}$ is the mean counts in the VOI to be quantified, $\:\text{t}$ is the time per projection [s], $\:\text{n}$ is the number of projections, $\:{V}_{voxel}$ is the voxel volume [mL], $\:A$ is the true total activity in the VOI [MBq], and $\:{V}_{VOI}$ is the volume of the VOI [mL]. The CF_homogeneous_ was calculated based on the activity present at scanning in a two liter cylindrical VOI centered in the homogeneous phantom. The CF_self_ was calculated based on the total activity present in the Jaszczak phantom at the start of each SPECT acquisition, and a VOI encompassing the whole phantom. Each Flash3D-reconstruction was processed to have the unit of counts per second per milliliter [cps/mL], and was thereafter quantified by dividing each voxel in the image with either the CF_homogeneous_ or CF_self_. Lastly, the quantified images [MBq/mL] were decay corrected to the injection time point.

The Broad Quantification-based quantification method directly quantifies the xSPECT-reconstruction in units of Bq/mL and decay corrects it to the injection time point, see supplementary information for information about the calibration procedure.

#### Homogeneity evaluation in cylindrical phantom

Based on the quantitative SPECT images of the cylindrical homogeneous phantom, the homogeneity of the reconstructions were evaluated through assessment of activity concentration profiles (ACPs) parallel to the phantom’s symmetry axis. Three voxel row profiles and two cylindrical VOIs were evaluated. The ACPs for the cylindrical VOIs were obtained by averaging the activity concentration values, for all voxels within a specified radius, per slice in the SPECT data. Furthermore, cumulative activity-volume histograms (cAVH) have been calculated for one VOI encompassing the whole phantom volume, and two cylindrical VOIs centered in and having the same length, but with a smaller radius (either 30 mm or 70 mm), as the cylindrical phantom. The cAVHs were qualitatively compared to the theoretical ideal cAVH for a homogeneous distribution, which is a step function. Images without post-reconstruction filtering and with a 15 mm FWHM Gaussian filter were compared in this analysis.

#### Activity recovery in Jaszczak phantom

The percentage of recovered activity was computed for each of the three SPECT quantification methods and each time point of the Jaszczak phantom measurements. All the voxels in a VOI encompassing the whole phantom, i.e. the same VOI as was used for the determination of CF_self_, was considered for this calculation.

#### Activity concentration recovery coefficient

The activity concentration recovery coefficients (ACRC) were calculated for the sphere VOIs, and the background VOI for each SPECT quantification method and for each time point of the Jaszczak phantom measurements. The ACRC was defined as$\:ACRC=\frac{A{C}_{quantifiedSPECT}}{{AC}_{true}},$

where$\:{\:AC}_{quantifiedSPECT}$ is the mean activity concentration of the sphere or background VOI in the quantified SPECT image [MBq/mL], and $\:{AC}_{true}$ is the true activity concentration in the same VOI [MBq/mL].

#### Recovery curve

Recovery curves that can be used for correcting images for losses related to partial volume effects (PVEs), i.e. ACRC as a function of sphere diameter *(d)*, were created. The ACRCs for the three repeated measurements at time point 10, 12, 13 and 14 were averaged for each quantification method and the dependency of the activity level was visualized. A 3-parameter *(a*,* b*,* c)* logistic function on the form

$$\:ACRC\left(d\right)=a\left(1-\frac{1}{1+{\left(\frac{d}{b}\right)}^{c}}\right)$$

was fitted to the data [13]. The *a-*coefficient represents the asymptote that the fitted curve is converging towards when increasing the spherical diameter of a hot object, indicating the theoretically highest ACRC possible for each quantification method and activity level.

## Results

### Conversion factors for SPECT quantification

**Fig. 2:**
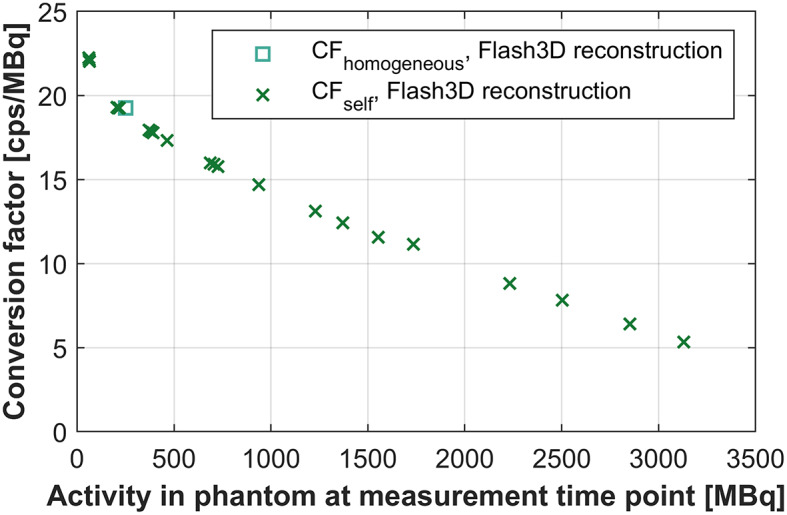
Conversion factors (CF) for two quantification methods utilizing the Flash3D reconstructions: CF_homogeneous_ and CF_self_. The CF_homogeneous_ has been measured and calculated for one time point (at 250 MBq) and the CF_self_ was calculated for each measurement of the Jaszczak phantom (total of 22 measurements)

Conversion factors used for quantifying the Flash3D-reconstructions were calculated based on the homogeneous phantom and for each measurement of the Jaszczak phantom, see Fig. 2. The value of CF_homogeneous_ (19.26 cps/MBq) is similar to the CF_self_ determined at 206–217 MBq (19.26–19.29 cps/MBq).

### Homogeneity cylindrical phantom

**Fig. 3:**
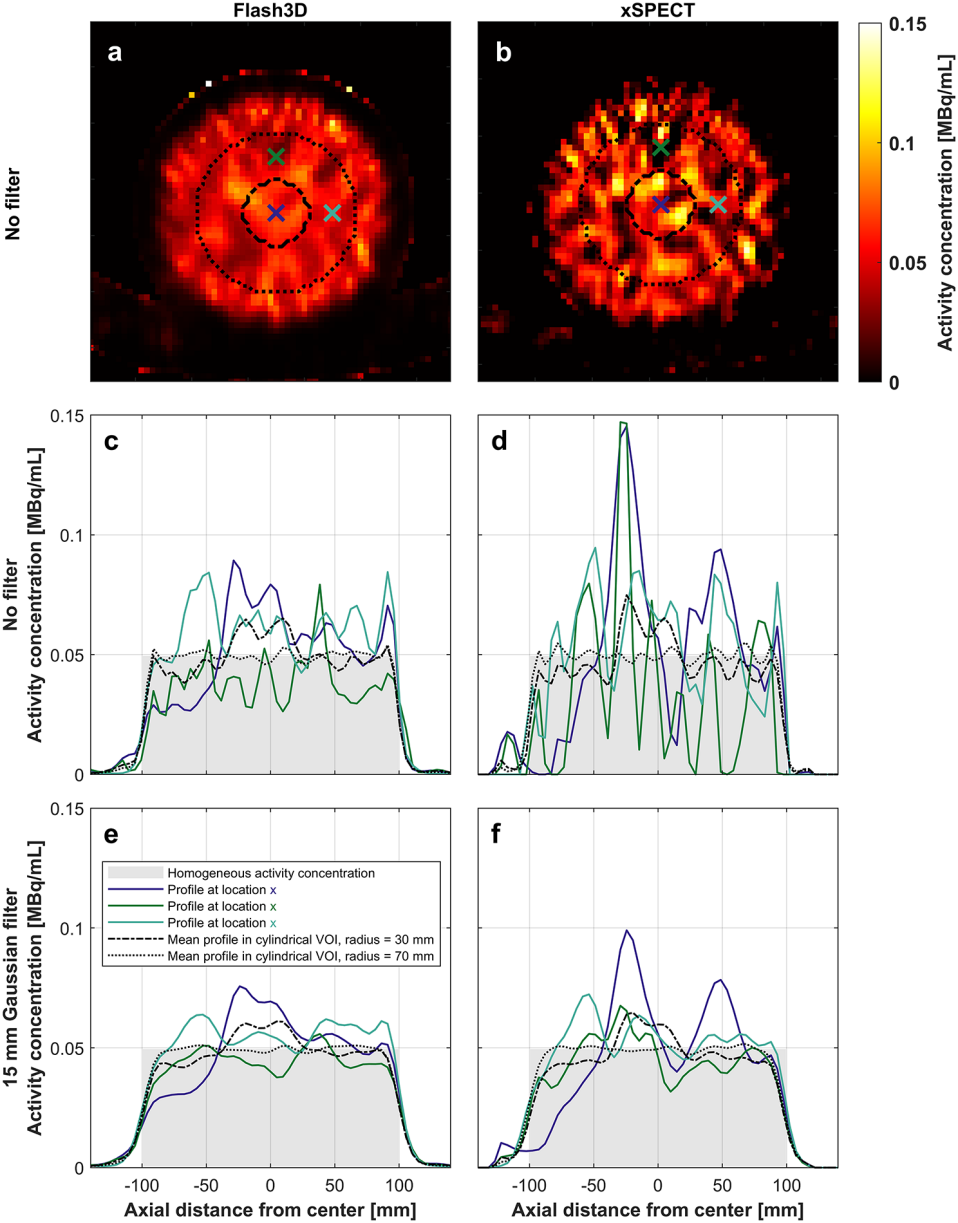
Visualization of the homogeneity in the homogeneous cylindrical phantom. A cross-section of the homogeneous cylindrical phantom is shown in A and B for unfiltered Flash3D and xSPECT-reconstructions, respectively. The locations and the VOIs considered for the visualized activity concentration profiles (ACPs) are also depicted in **a** and **b**, the profiles are orthogonal to the cross-sectional plane. The ACPs for the datasets shown in **a** and **b**, are plotted in **c** and **d**. In **e** and **f**, the same ACPs are shown, but for filtered versions of the datasets in **a** and **b**. The distinctive appearance of the voxel row ACPs (solid lines; purple, green, cyan) is highly spatially dependent. The ACP that has been average over a radius of 70 mm is considered to match the true homogeneous activity concentration

The ACPs for the cylindrical homogeneous phantom can be found in Fig. 3. The locations of the voxel profiles and volumes are visualized in Fig. 3a, b and the profiles in Fig. 3c-f. The ACPs for the displayed voxel rows of the unfiltered Flash3D-reconstruction vary between 53% and 181% of the AC_true_ within the phantom (a margin of 30 mm from the edge was considered), and for the xSPECT-reconstruction the corresponding value vary between 0% and 297% (Fig. 3c, d). After filtering this variation has been reduced to 62–153% and 52–200% of AC_true_, for Flash3D and xSPECT respectively (Fig. 3e, f). The ACPs averaged over a radius of 30 mm is varying between 77% and 132% of AC_true_ for the unfiltered Flash3D, and 75% and 151% for the unfiltered xSPECT-reconstructions. This is reduced to 85–123% and 87–130% for the filtered Flash3D and xSPECT data, respectively. The ACPs averaged over a radius of 70 mm can be considered to conform to AC_true_ as the deviation is within ± 10% (93–107% unfiltered Flash3D, 91–109% unfiltered xSPECT, 96–103% filtered Flash3D, 95–104% filtered xSPECT).

**Fig. 4:**
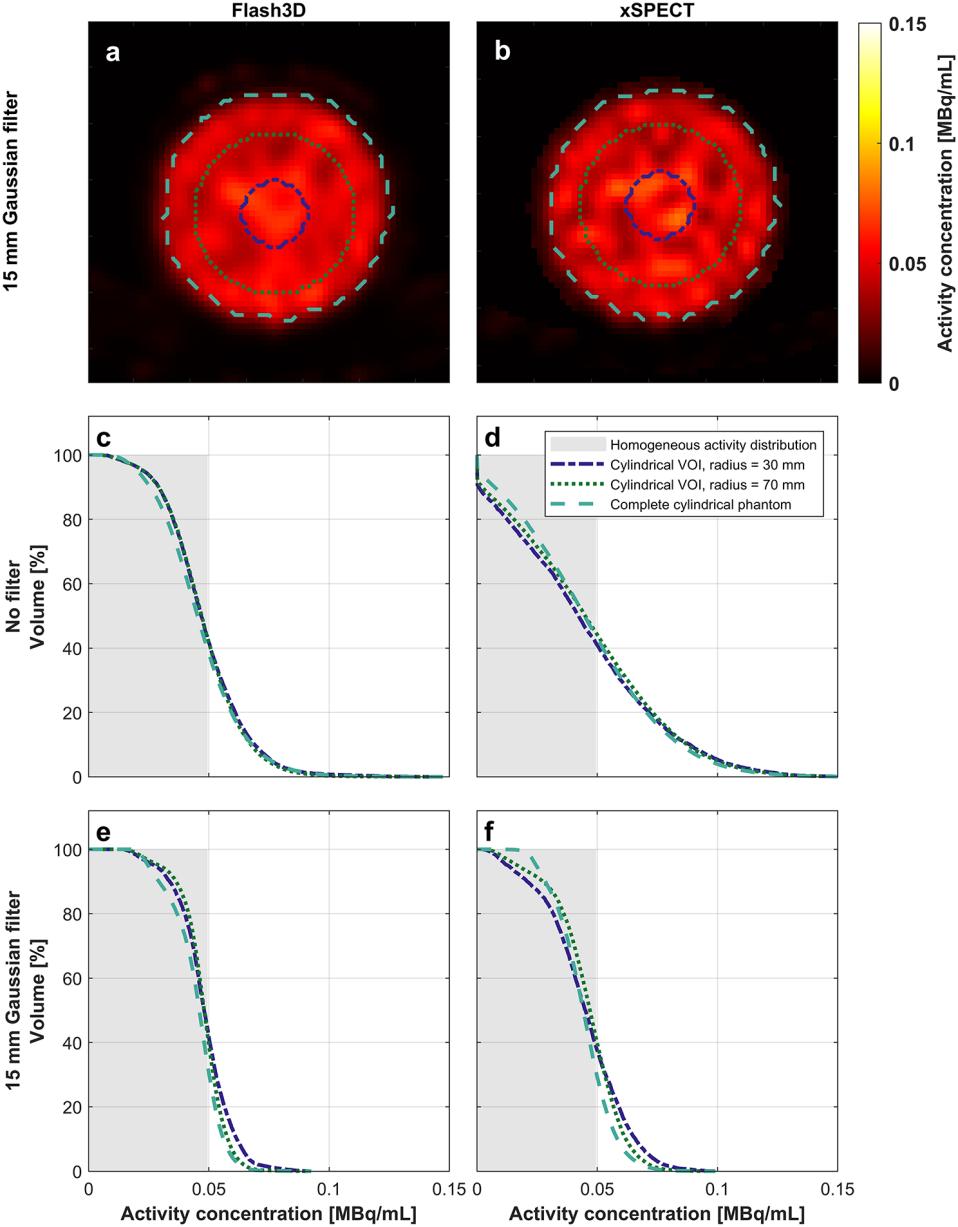
(**a**,**b**) A cross-section of the homogeneous cylindrical phantom for filtered Flash3D and xSPECT-reconstructions (same slice as in Fig. 3), respectively, including the cross-sections of the VOIs considered in the cumulative activity-volume histograms (cAVHs). (**c-f**) cAVHs of the homogeneous phantom for the unfiltered data (**c**,**d**) and filtered data (**e**,**f**).The accuracy of the cAVH-curves, improves for the filtered the data compared to the unfiltered data. Even in the most accurate cAVH, cylindrical VOI with a radius of 70 mm (green dotted line) in **e**, the activity concentration is underestimated for 58% of the volume and overestimated for 42% of the volume

The cAVH-curves for the cylindrical homogeneous phantom can be found in Fig. 4. The cross-sections of the VOIs considered for this analysis are visualized in Fig. 4a, b and the corresponding curves can be found in Fig. 4c-f. None of the cAVH-curves follow the step function (indicated in grey), but there is better agreement for the filtered data (Fig. 4e, f) compared to the unfiltered data (Fig. 4c, d). In Fig. 4d, the heterogeneous activity concentration distribution resulting from the unfiltered xSPECT-reconstruction (visualized in Fig. 3b, d) is shown. According to this graph more than 5% of the total phantom volume holds less than 0.001 MBq/mL, and more than 4% of the volume contains more than two times the actual activity concentration (0.098 MBq/mL).

### Activity recovery

**Fig. 5:**
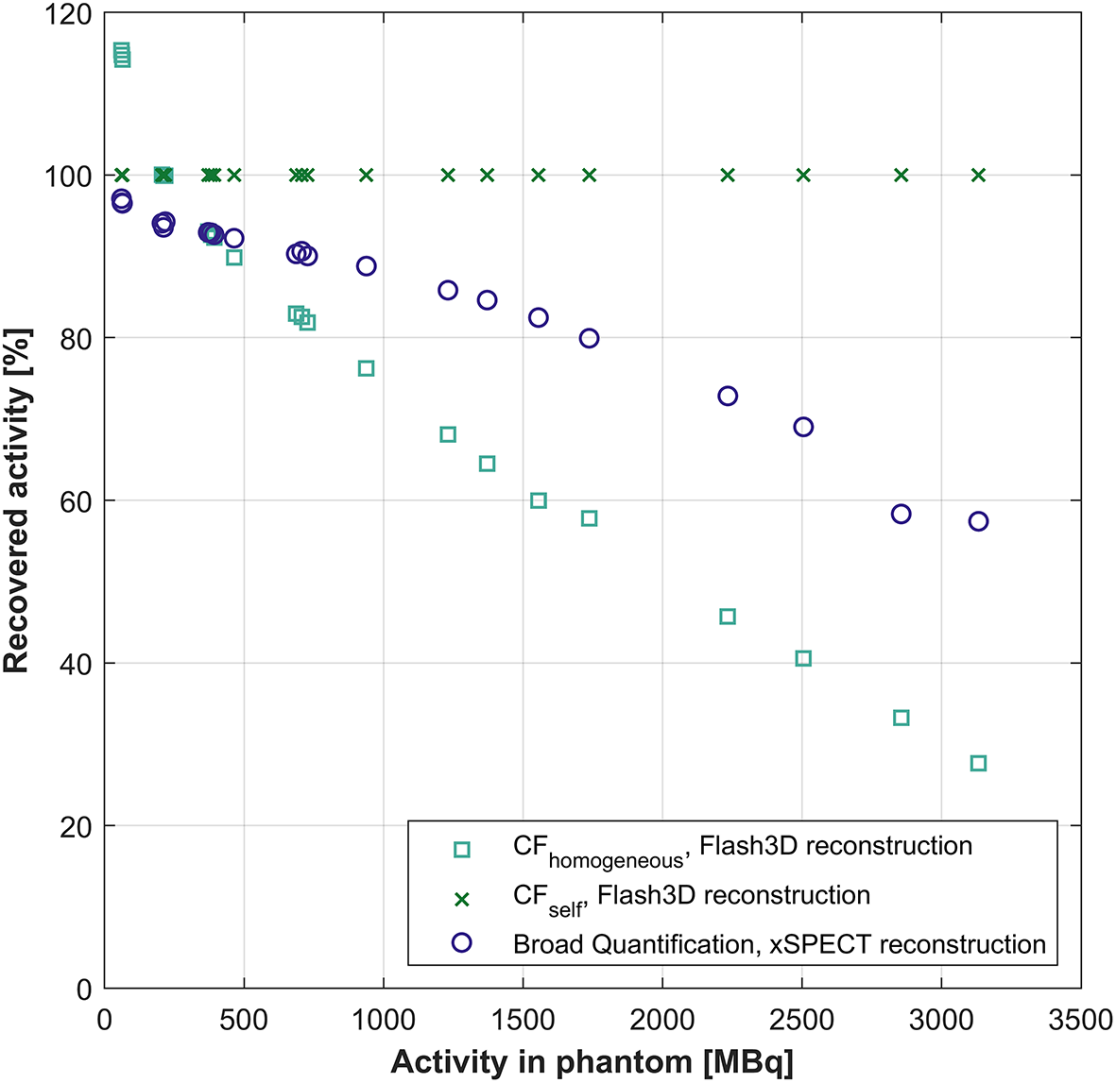
Percentage of recovered activity in the Jaszczak phantom, based on the quantified SPECT/CT images for each measurement time point and quantification method. For CF_homogeneous_, the recovery is 28–115% depending on the activity level, and for Broad Quantification it is 57–97%. The recovery for CF_self_ is always 100%

The percentage of the recovered activity for each quantification method and measurement of the Jaszczak phantom is visualized in Fig. 5. In general, CF_homogeneous_ underestimates the amount of activity for measurements performed at high activities and overestimates the amount of activity for measurements at low activities; 28% of the activity is recovered at time point 1 (3130 MBq), and 115% of the activity is recovered at time point 14 (61 MBq). As a direct result of the definition of the CF_self_, the recovered activity for this method is always 100%, independent of the activity level. Similarly to CF_homogeneous_, the accuracy of the Broad Quantification method is also hampered at the measurements performed at high activities, but in contrast to CF_homogeneous_ this method does not overshoot the recovered activity at low activities; 57% of the activity is recovered at time point 1, and 97% of the activity is recovered at time point 14.

For CF_homogeneous_, only the data acquired with 206–393 MBq present in the phantom results in a maximum deviation of 10% from the true total amount of recovered activity. For the Broad Quantification a maximum deviation of 10% is obtained for data acquired at 61–464 MBq.

### Activity concentration recovery coefficient

**Fig. 6:**
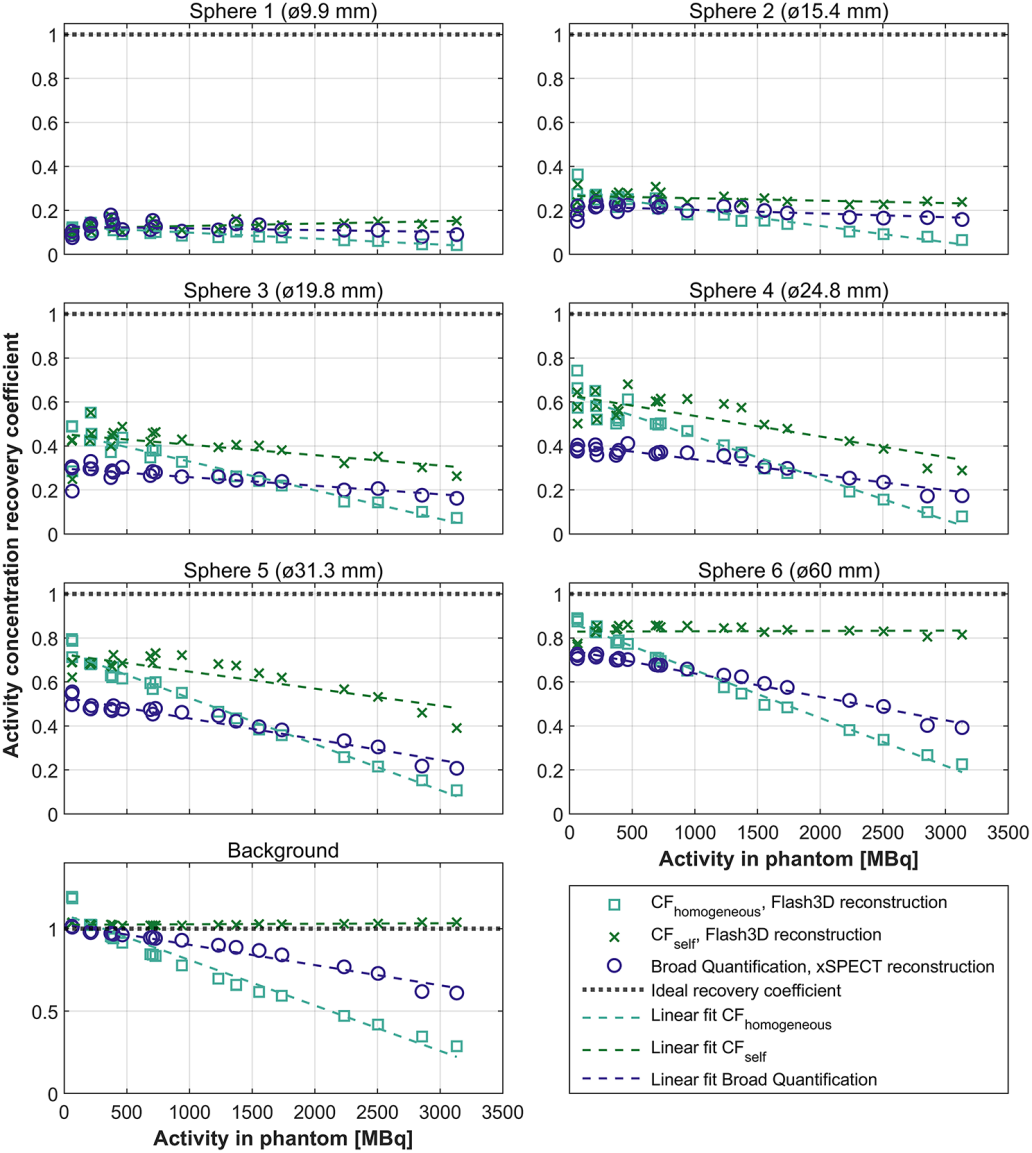
Activity concentration recovery coefficients, as a function of total activity in the Jaszczak phantom at the measurement time point, for the three quantification methods. An ACRC = 1 is considered ideal (dotted line). A linear model (dashed lines) has been fitted to the data to visualize the trend

The ACRC for each quantification method as a function of total activity at each measurement time point, for each VOI in the Jaszczak phantom, can be found in Fig. 6. A linear model has been fitted to the ACRC datapoints to indicate the trend over the full range of measured activities. In general, CF_self_ has the highest ACRCs for the spheres, with the exception of the measurements at the lowest measured activity (time point 14; 61–64 MBq) where the CF_homogeneous_ overestimates the recovered activity in the whole image, which also results in an overshoot in the ACRC for all VOIs. The Broad Quantification method performs better than CF_homogeneous_ at high activities (above approximately 2000 MBq), but worse at the lower activities (below approximately 1000 MBq). One contributing factor for this is the lower contrast recovery that is achieved when utilizing the xSPECT-reconstruction method compared to the Flash3D-reconstruction method, see supplementary information. CF_self_ slightly overestimates the ACRC in the background VOI (by 2–4%) for all measurement time points because of the imperfect contrast recovery, see supplementary information, in combination with the definition of CF_self_ which forces the true activity to be distributed in the image.

The impact of lower detection efficiency at high activities, as a result of higher dead-time (see Table 1), is also visible in Fig. 6. The ACRC for Sphere 6 and CF_self_ is however relatively stable across all measurement time points (range: 0.76–0.86), but drops slightly at the lower end of the measured activities, probably because of the lower count rate statistics.

### Recovery curves

**Fig. 7:**
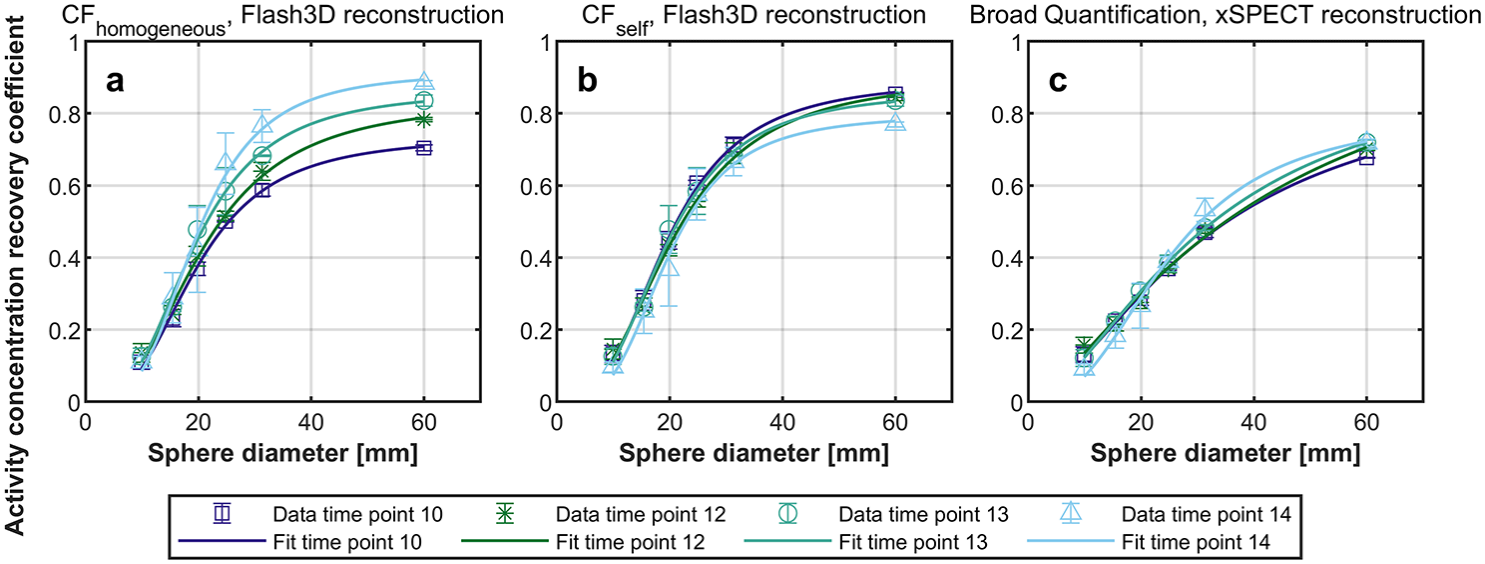
Activity concentration recovery coefficients (ACRCs) as a function of sphere diameter, for each of the three quantification methods (**a**-**c**). The ACRCs from the three repeated measurements, of time point 10, 12, 13, and 14 (687–727 MBq, 371–393 MBq, 206–217 MBq, and 61–64 MBq), have been averaged, and the mean and standard deviation are plotted in the graphs. Recovery curves (3-parameter logistic functions) have been fitted to the data, with R^2^ > 0.99 for all curves

The ACRCs for time point 10, 12, 13 and 14, as a function of sphere diameter, can be seen in Fig. 7. The data has been averaged over the three consecutive repetitions for each of these time points, and are presented by their mean and standard deviation. Recovery curves (3-parameter logistic function) have been fitted to the data and are also shown in the graph. The coefficient of determination (R^2^) was > 0.99 for all fitted curves. The asymptote value from this fit is presented in Table 2.

**Table 2:** The *a*-coefficient from the fitted recovery curves (3-parameter logistic functions) representing the asymptote that the curve would converge to with increasing hot sphere diameter

	CF_homogeneous_, Flash3D-reconstruction	CF_self_, Flash3D-reconstruction	Broad Quantification, xSPECT-reconstruction
Time point 10	0.74	0.89	0.92
Time point 12	0.84	0.90	1.10
Time point 13	0.86	0.86	0.96
Time point 14	0.91	0.80	0.80

## Discussion

In the presented study we have evaluated the accuracy of three SPECT quantification methods for ^166^Ho across a wide activity range. The Flash3D-reconstruction algorithm combined with the self-calibration quantification method (CF_self_) resulted in the most accurate activity recovery (Fig. 5) and the highest sphere recovery over the whole activity range (Fig. 6).

No linear relationship between the measured counts per second in the photopeak energy window, and the activity present in the phantom at scanning has been found for our data, see counting rate performance in the Supplementary information. This is due to nonlinearities in the measured count rate with respect to the scanned activity level, dead-time effects and pulse pile-up [4, 21]. This indicates that a conversion factor based on a single ^166^Ho activity level (CF_homogeneous_) would not be suitable to use for quantification of data at different ^166^Ho activity levels (Fig. 2). Furthermore, we have demonstrated that applying CF_homogeneous_ results in significant underestimation of the recovered activity at high activity levels and overestimation of the recovered activity at low activity levels (Fig. 5). In contrast to a recent guideline [12], we can therefore not recommend this methodology for quantifying ^166^Ho-SPECT/CT images for clinical purposes. Additionally, the absolute quantification method (Broad Quantification) also gravely underestimates the recovered activity at high ^166^Ho activity levels, but is more accurate than CF_homogeneous_ at low activity levels (Fig. 5).

In the homogeneous phantom (Fig. 3), we see that the unfiltered xSPECT-reconstruction contains high levels of noise and is therefore not suitable for clinical evaluation of homogeneous activity distributions. After filtering, this noise is reduced but the variation in the voxel values is still large. The noise levels in the unfiltered Flash3D-reconstruction is comparable to the filtered xSPECT-reconstruction, however both reconstruction methods (independent of the filtering parameters) produce images in which the voxel values are highly location dependent and there is hence a large uncertainty in the measured voxel value.

Examining the cAVHs of the cylindrical homogeneous phantom (Fig. 4), we see that the imaged heterogeneity (i.e. the uncertainty in the measured voxel value) is spread out through the whole phantom as the curves for the different VOIs, for each separate reconstruction method and filtering parameter, are very similar. Considering that the true activity concentration in the cylindrical phantom is uniform and that the ^166^Ho-SPECT/CT images are inherently heterogeneous, neither of the investigated reconstructions methods can be deemed to produce images which are suitable for voxel-level assessment of homogeneous distributions [22]. An additional drawback with utilizing activity- or dose-volume histograms to assess activity-/dose distributions for clinical assessment is that no spatial information is retained. Considering that microsphere distributions after TARE are inherently heterogeneous [23] and that this information is lost in a dose volume histogram, this metric should be used with caution in radionuclide therapy when evaluating the tumor coverage [24].

From the Jaszczak phantom measurements, we verified the quantification accuracy over a large activity range, determined ACRCs, and constructed the associated recovery curves. Figure 6 indicates poor recovery (ACRC < 0.36) of the two smallest spheres, regardless of quantification method or activity level, making small lesion dosimetry challenging with ^166^Ho-SPECT/CT imaging. The visibility of the smallest spheres appears to be worse for ^166^Ho-SPECT than for other radionuclides [25–27]. The measured ACRCs are however not only dependent on quantification method, radioisotope, and activity level, but also on sphere position within the phantom, $\:\text{S}:\text{B}{\text{G}}_{\text{r}\text{a}\text{t}\text{i}\text{o}}$, PVE compensations, and delineation method. Two of the most frequently used delineation methods are anatomical- (CT-/MRI-based), as used in this study, and threshold-based [28]. Considering all these factors, it is therefore not always easy or even appropriate to directly compare the ACRCs from different studies.

When performing clinical dosimetry, especially when imaging small lesions, the recovery curves in Fig. 7 could be used to correct for PVEs [13]. This type of partial volume correction (PVC) method is region-based, meaning that voxel-level evaluation of the absorbed dose distribution must not be carried out after this correction has been applied [29]. In Fig. 7a (CF_homogeneous_), the recovery curve shifts upwards as the activity in the phantom decreases (later time point). This shift is however not a result of better image quality or higher quantitative accuracy at a later time point, but a result of the bias in the recovered activity that is introduced when CF_homogeneous_ is used to quantify the data. In contrast, Fig. 7b (CF_self_) shows that the recovery curves are comparable for time points 10, 12, and 13, but that the curve is shifted downwards for time point 14 as a result of lower count statistics in the acquired data (i.e. higher image noise) and hence lower ACRCs. Unless this lower count statistics can be compensated for by increasing the total scan time, this indicates that clinical dosimetry should not be carried out based on ^166^Ho-SPECT imaging with too low activity. The data in Fig. 7c (Broad Quantification) has an overall different shape compared to Fig. 7a, b, with lower ACRCs and these recovery curves are further from convergence. The asymptote of the fitted recovery curves, see Table 2, is nevertheless predicting a higher ACRC for the Broad Quantification method (range: 0.80–1.10) than for the other two quantification methods, but for sphere diameters (hot object size) which are larger than what is currently used in this Jaszczak phantom. For example, when extrapolating the recovery curve for the Broad Quantification method and time point 13 (not shown in the figure), an ACRC of 0.90 (10% loss) is only reached for a spherical diameter of 157 mm which is larger than the average normal liver size [30].

Even though quantifying the ^166^Ho-SPECT/CT images using CF_self_ is the most robust for the total activity recovery with respect to the imaged activity level (Fig. 5), recovery curve PVC is still dependent on the activity level (independent of which quantification method is used), and applying this correction method for clinical data is therefore a rather complex endeavor. Another drawback with recovery curve PVC is that when it is used in combination with CF_self_-based quantification, then the total amount of activity in the patient would be overestimated.

The activity level significantly affects the ^166^Ho-SPECT/CT image quality. Too high activity leads to limited quantification accuracy due to dead-time effects, while reduced count statistics can be an issue at low activities. Clinically administered ^166^Ho activities can exceed 8 GBq [3, 31, 32], and could potentially be even higher when personalized image-guided dose escalation is developed [33]. When personalizing the treatment, the amount of administered activity would not always be known before the start of the procedure and it could be difficult to determine the optimal time point for the SPECT/CT scan upfront. There is therefore a clinical need for limiting the negative effects of a high dead-time on the image quality. TrueCalc has previously been shown to improve the accuracy of the measured activity concentration for technetium-99m (^99m^Tc) and lutetium-177 (^177^Lu) scanned at high count rates [20]. In this study we were however not able to compare the performance of the Broad Quantification with and without TrueCalc (when TrueCalc has been installed on a clinical SPECT/CT scanner it is always active when acquiring xSPECT data in ‘Quantitative Acquisition’’-mode), and can therefore not investigate the effect of TrueCalc for ^166^Ho-SPECT/CT imaging as a dead-time correction method. Other dead-time correction methods, based on the acquisition of the wide-spectrum count rate, have been suggested for ^177^Lu [34, 35]. However, because some of the high-energy photons of ^166^Ho are lying outside of the measurable energy interval (up to about 700 keV, depending on the SPECT/CT scanner), these methods did not seem appropriate for ^166^Ho. Our data suggests that performing ^166^Ho-SPECT/CT imaging with approximately 200–400 MBq would ensure < 10% error in the total amount of recovered activity, independent of the quantification method used.

To reliably use the CF_self_ for quantification of clinical ^166^Ho-SPECT/CT images, it is important to monitor the activity administered to the patient. The administered activity should be based on a pre-treatment measurement of the activity in the v-vial in a calibrated dose calibrator, and corrected based on the a post-treatment measurement of the residual activity in the v-vial and the delivery set. If such a measurement is not possible, it is reasonable to assume a residual activity of 3.5–6.9% after a TARE procedure with ^166^Ho microspheres [36]. Additionally, the value of the activity used in the determination of CF_self_ should be corrected for the fraction of activity that reaches the targeted region or organ (in cases of shunting). This can be done either based on the ^166^Ho-SPECT/CT image or on planar scintigraphy [37]. Another point of concern in clinical SPECT-based dosimetry for TARE is the degenerative effect of respiratory motion on the images, as this can introduce motion induced spill-in and spill-out of counts, as well as misalignment between the nuclear medicine image and the anatomical image [15]. Respiratory induced liver motion alone has been shown to on average decrease the activity recovery and tumor/non-tumor ratios with 24% points in a simulation study of a ^99m^Tc digital phantom [38]. One option for mitigating this effect is to apply dilation correction of tumor VOIs when assessing the tumor doses [4, 7]. Preferably however, the projection data should be corrected for motion artifacts before being reconstructed [39], but this technique is not available on all SPECT/CT scanners. An even better option for eliminating artifacts originating from respiratory motion would be to acquire the imaging data in breath hold. The acquisition time for ^166^Ho-SPECT is currently too long (approximately 20 min acquisition in our clinical protocol) to be able to efficiently include respiratory gating in the clinical workflow. As an alternative to conventional nuclear imaging, MR imaging acquired in breath hold could be used to measure the distribution of ^166^Ho microspheres after TARE [9, 10]. MRI has the advantage of being much faster than SPECT (2–3 min for acquisition of a whole liver), as well as having higher resolution (in-plane resolution 2 × 2 mm^2^, slice thickness 4 mm), and being quantitative. Additionally, MRI can also be used for image guidance during the TARE procedure [31].

## Conclusion

In conclusion, for clinical dosimetry we recommend to reconstruct the SPECT images using the Flash3D algorithm and quantify the images based on a self-calibration CF. For this it is essential to accurately measure the activity that has been administered to the patient, using a calibrated dose calibrator. The injected activity should also be corrected for possible shunting from the targeted region. This fraction can be estimated either on the ^166^Ho-SPECT/CT image or on planar scintigraphy, and should be used to adjust the activity present in the calculation of the CF_self_. 

## Electronic supplementary material

Below is the link to the electronic supplementary material.

Supplementary Material 1

## Data Availability

The data that were generated and analyzed in this study are available from the corresponding author on reasonable request.

## Abbreviations

^99m^Tc: Technetium-99m
^166^Ho: Holmium-166
^177^Lu: Lutetium-177
AC: Activity concentration
ACP: Activity concentration profile
ACRC: Activity concentration recovery coefficients
cAVH: Cumulative activity-volume histograms
CF: Conversion factor
CF_homogeneous_: Scanner-specific conversion factor based on homogeneous phantom
CF_self_: Self-calibration conversion factor
CT: Computed tomography
FWHM: Full width at half maximum
MELP: Medium Energy Low Penetration collimators
MRI: Magnetic resonance imaging
OSCG: Ordered subset conjugate gradient
OSEM: Ordered subset expectation maximization
PVC: Partial volume correction
PVE: Partial volume effect
S:BG_ratio_: Sphere-to-background activity concentration ratio
SPECT: Single photon emission computed tomography
TARE: Transarterial radioembolization
VOI: Volumes-of-interest

## References

[1] Klaassen NJM, Arntz MJ, Gil Arranja A, Roosen J, Nijsen JFW. The various therapeutic applications of the medical isotope holmium-166: a narrative review. EJNMMI Radiopharm Chem. 2019;4:1–26. 31659560 10.1186/s41181-019-0066-3 PMC6682843

[2] Bakker RC, Van Es RJJ, Rosenberg AJWP, Van Nimwegen SA, Bastiaannet R, De Jong HWAM, et al. Intratumoral injection of radioactive holmium-166 microspheres in recurrent head and neck squamous cell carcinoma: preliminary results of first use. Nucl Med Commun. 2018;39:213–21. 29309367 10.1097/MNM.0000000000000792 PMC5815636

[3] Smits MLJ, Nijsen JFW, van den Bosch MAAJ, Lam MGEH, Vente MAD, Mali WPTM, et al. Holmium-166 radioembolisation in patients with unresectable, chemorefractory liver metastases (HEPAR trial): a phase 1, dose-escalation study. Lancet Oncol. 2012;13:1025–34. 22920685 10.1016/S1470-2045(12)70334-0

[4] Stella M, Braat AJAT, Lam MGEH, de Jong HWAM, van Rooij R. Gamma camera characterization at high holmium-166 activity in liver radioembolization. EJNMMI Phys. 2021;8. 10.1186/s40658-021-00372-9 PMC7925770 33651253

[5] Bé M-M, Chisté V, Dulieu C. Bureau International des Poids et Mesures. Table of radionuclides. Vol. 2, A = 151 to 242 [Internet]. Bureau International des Poids et Mesures; 2004 [cited 2024 Mar 13]. https://www.bipm.org/documents/20126/53814638/Monographie+BIPM-5+-+Volume+2+%282004%29.pdf/047c963d-1f83-ab5b-7983-744d9f48848a

[6] Deslattes RD, Kessler EG, Indelicato P, de Billy L, Lindroth E, Anton J. X-ray transition energies: new approach to a comprehensive evaluation. Rev Mod Phys [Internet]. 2003;75:35–99. 10.18434/T4859Z

[7] Smits MLJ, Dassen MG, Prince JF, Braat AJAT, Beijst C, Bruijnen RCG et al. The superior predictive value of 166Ho-scout compared with 99mTc-macroaggregated albumin prior to 166Ho-microspheres radioembolization in patients with liver metastases. Eur J Nucl Med Mol Imaging. 2019. 10.1007/s00259-019-04460-y PMC7075844 31399801

[8] Roosen J, Klaassen NJM, Westlund Gotby LEL, Overduin CG, Verheij M, Konijnenberg MW, et al. To 1000 gy and back again: a systematic review on dose-response evaluation in selective internal radiation therapy for primary and secondary liver cancer. Eur J Nucl Med Mol Imaging. 2021;48:3776–90. 33839892 10.1007/s00259-021-05340-0 PMC8484215

[9] Van De Maat GH, Seevinck PR, Elschot M, Smits MLJ, De Leeuw H, Van Het Schip AD, et al. MRI-based biodistribution assessment of holmium-166 poly(L-lactic acid) microspheres after radioembolisation. Eur Radiol. 2013;23:827–35. 23014797 10.1007/s00330-012-2648-2 PMC3563959

[10] Roosen J, van Wijk MWM, Westlund Gotby LEL, et al. Improving MRI-based dosimetry for holmium-166 transarterial radioembolization using a nonrigid image registration for voxelwise *ΔR*_2_* calculation. Med Phys. 2023;50:935–46. 10.1002/mp.16014 10.1002/mp.16014 36202392

[11] Sjögreen-Gleisner K, Flux G, Bacher K, Chiesa C, de Nijs R, Kagadis GC, et al. EFOMP policy statement 19: Dosimetry in nuclear medicine therapy – molecular radiotherapy. Phys Med. 2023;116:1–5. 10.1016/j.ejmp.2023.103166 37926641

[12] Dickson JC, Armstrong IS, Gabiña PM, Denis-Bacelar AM, Krizsan AK, Gear JM et al. EANM practice guideline for quantitative SPECT-CT. Eur J Nucl Med Mol Imaging [Internet]. 2023;50:980–95. https://link.springer.com/10.1007/s00259-022-06028-9 10.1007/s00259-022-06028-9 PMC9931838 36469107

[13] Gear JI, Cox MG, Gustafsson J, Gleisner KS, Murray I, Glatting G, et al. EANM practical guidance on uncertainty analysis for molecular radiotherapy absorbed dose calculations. Eur J Nucl Med Mol Imaging. 2018;45:2456–74. 30218316 10.1007/s00259-018-4136-7 PMC6208822

[14] Dewaraja YK, Frey EC, Sgouros G, Brill AB, Roberson P, Zanzonico PB, et al. MIRD pamphlet 23: quantitative SPECT for patient-specific 3-dimensional dosimetry in internal radionuclide therapy. J Nucl Med. 2012;53:1310–25. 22743252 10.2967/jnumed.111.100123 PMC3465844

[15] Chiesa C, Sjogreen-Gleisner K, Walrand S, Strigari L, Flux G, Gear J et al. EANM dosimetry committee series on standard operational procedures: a unified methodology for 99mTc-MAA pre- and 90Y peri-therapy dosimetry in liver radioembolization with 90Y microspheres. EJNMMI Phys. 2021;8. 10.1186/s40658-021-00394-3 PMC8589932 34767102

[16] Elschot M, Smits MLJ, Nijsen JFW, Lam MGEH, Zonnenberg BA, Van Den Bosch MAAJ et al. Quantitative Monte Carlo-based holmium-166 SPECT reconstruction. Med Phys. 2013;40. 10.1118/1.4823788 24320461

[17] Vija A. Introduction to xSPECT* Technology: Evolving Multi-modal SPECT to Become Context-based and Quantitative. White Pap [Internet]. 2013;1–28. http://www.healthcare.siemens.com/siemens_hwem-hwem_ssxa_websites-context-root/wcm/idc/groups/public/@global/@imaging/@molecular/documents/download/mdax/ote3/~edisp/xspect_technical_white_paper-00957532.pdf

[18] GE Healthcare. White paper: NM Quantification Q.Metrix for SPECT/CT Package.

[19] Vija AH. White paper: Characteristics of the xSPECT reconstruction method [Internet]. 2017. https://marketing.webassets.siemens-healthineers.com/1800000004275045/7489c11bd57e/MI-2735_NS_RECONSTRUCTION_WP.final_1800000004275045.pdf

[20] Vija AH, Zeintl J. Quantitative Assessment of Accuracy and Precision for High Count Rate Spect Imaging of 99mTc and 177Lu. Annu Congr Eur Assoc Nucl Med Oct 13–7, 2018 Düsseldorf, Ger. 2018. p. S16.

[21] Ritt P, Vija H, Hornegger J, Kuwert T. Absolute quantification in SPECT. Eur J Nucl Med Mol Imaging. 2011;38:69–77. 10.1007/s00259-011-1770-8 21484383

[22] Tran-Gia J, Salas-Ramirez M, Lassmann M. What you see is not what you get: on the accuracy of Voxel-based dosimetry in molecular radiotherapy. J Nucl Med. 2020;61:1178–86. 31862802 10.2967/jnumed.119.231480 PMC7413234

[23] Pillai KM, Mckeever PE, Knutsen CA, Terrio PA, Prieskorn DM, Ensminger WD. Microscopic analysis of arterial Microsphere distribution in rabbit liver and hepatic VX2 tumor. Sel Cancer Ther. 1991;7:39–48. 1754728 10.1089/sct.1991.7.39

[24] Drzymala RE, Mohan R, Brewster L, Chu J, Goitein M, Harms W et al. Dose-volume histograms. Int J Radiat Oncol Biol Phys [Internet]. 1991;21:71–8. https://linkinghub.elsevier.com/retrieve/pii/0360301691901684 10.1016/0360-3016(91)90168-4 2032898

[25] Peters SMB, Meyer Viol SL, van der Werf NR, de Jong N, van Velden FHP, Meeuwis A et al. Variability in lutetium-177 SPECT quantification between different state-of-the-art SPECT/CT systems. EJNMMI Phys. 2020;7. 10.1186/s40658-020-0278-3 PMC7013023 32048097

[26] Zeintl J, Vija AH, Yahil A, Hornegger J, Kuwert T. Quantitative accuracy of clinical 99mTc SPECT/CT using ordered-subset expectation maximization with 3-dimensional resolution recovery, attenuation, and scatter correction. J Nucl Med. 2010;51:921–8. 20484423 10.2967/jnumed.109.071571

[27] Collarino A, Pereira Arias-Bouda LM, Valdés Olmos RA, van der Tol P, Dibbets-Schneider P, de Geus-Oei LF, et al. Experimental validation of absolute SPECT/CT quantification for response monitoring in breast cancer. Med Phys. 2018;45:2143–53. 29572848 10.1002/mp.12880

[28] Lam M, Garin E, Palard-Novello X, Mahvash A, Kappadath C, Haste P et al. Direct comparison and reproducibility of two segmentation methods for multicompartment dosimetry: round robin study on radioembolization treatment planning in hepatocellular carcinoma. Eur J Nucl Med Mol Imaging [Internet]. 2023;51:245–57. 10.1007/s00259-023-06416-9 10.1007/s00259-023-06416-9 PMC10684706 37698645

[29] Erlandsson K, Buvat I, Pretorius PH, Thomas BA, Hutton BF. A review of partial volume correction techniques for emission tomography and their applications in neurology, cardiology and oncology. Phys Med Biol. 2012;57. 10.1088/0031-9155/57/21/R119 23073343

[30] Wolf DC. Evaluation of the Size, Shape, and Consistency of the Liver. In: Walker H, Hall W, Hurst J, editors. Clin Methods Hist Phys Lab Exam [Internet]. 3rd editio. Boston: Butterworth Publishers; 1990. http://www.ncbi.nlm.nih.gov/pubmed/21250261 21250261

[31] Roosen J, Westlund Gotby LEL, Arntz MJ, Fütterer JJ, Janssen MJR, Konijnenberg MW et al. Intraprocedural MRI-based dosimetry during transarterial radioembolization of liver tumours with holmium-166 microspheres (EMERITUS-1): a phase I trial towards adaptive, image-controlled treatment delivery. Eur J Nucl Med Mol Imaging [Internet]. 2022;49:4705–15. 10.1007/s00259-022-05902-w 10.1007/s00259-022-05902-w PMC9606012 35829749

[32] Smits MLJ, Elschot M, Van Den Bosch MAAJ, Van De Maat GH, Van Het Schip AD, Zonnenberg BA, et al. In vivo dosimetry based on SPECT and MR imaging of 166Ho- microspheres for treatment of liver malignancies. J Nucl Med. 2013;54:2093–100. 24136931 10.2967/jnumed.113.119768

[33] Climical Trial - Radboud University Medical Center. NCT05609448 MRI-guided Holmium-166 Radioembolization (EMERITUS-2) [Internet]. 2022 [cited 2024 Jan 22]. https://clinicaltrials.gov/study/NCT05609448?locStr=Nijmegen

[34] Desy A, Bouvet GF, Lafrenière N, Zamanian A, Després P, Beauregard JM. Impact of the dead-time correction method on quantitative 177Lu-SPECT (QSPECT) and dosimetry during radiopharmaceutical therapy. EJNMMI Phys [Internet]. 2022;9. 10.1186/s40658-022-00484-w 10.1186/s40658-022-00484-w PMC9385894 35976503

[35] Frezza A, Desport C, Uribe C, Zhao W, Celler A, Després P, et al. Comprehensive SPECT/CT system characterization and calibration for 177Lu quantitative SPECT (QSPECT) with dead-time correction. EJNMMI Phys. 2020;7:1–22. 32060777 10.1186/s40658-020-0275-6 PMC7021856

[36] Drescher R, Seifert P, Gühne F, Aschenbach R, Kühnel C, Freesmeyer M. Radioembolization with Holmium-166 polylactic acid microspheres: distribution of residual activity in the Delivery Set and Outflow Dynamics during Planning and Treatment procedures. J Endovasc Ther. 2021;28:452–62. 33629598 10.1177/1526602821996719 PMC8129462

[37] Stella M, Braat AJAT, van Rooij R, de Jong HWAM, Lam MGEH. Holmium-166 Radioembolization: Current Status and Future Prospective. Cardiovasc Intervent Radiol [Internet]. 2022; 10.1007/s00270-022-03187-y 10.1007/s00270-022-03187-y PMC9626412 35729423

[38] Bastiaannet R, Viergever MA, De Jong HWAM. Impact of respiratory motion and acquisition settings on SPECT liver dosimetry for radioembolization. Med Phys. 2017;44:5270–9. 28736826 10.1002/mp.12483

[39] Sanders JC, Ritt P, Kuwert T, Vija AH, Maier AK. Fully Automated Data-Driven Respiratory Signal extraction from SPECT images using Laplacian Eigenmaps. IEEE Trans Med Imaging. 2016;35:2425–35. 27295657 10.1109/TMI.2016.2576899

