# Supplementary material for: Accuracy of holmium-166 SPECT/CT quantification over a large range of activities
Source: EJNMMI Phys. 2024 Sep 26;11:78. doi: 10.1186/s40658-024-00683-7 (PMC11427639; doi:10.1186/s40658-024-00683-7)
Supplement: Supplementary file 1 — Supplementary Material 1 [file 40658_2024_683_MOESM1_ESM.docx]

## Supplementary information for:

## Accuracy of holmium-166 SPECT/CT quantification over a large range of activities

EJNMMI Physics

Lovisa E. L. Westlund Gotby^1*^, Daphne Lobeek^1†^, Joey Roosen^1†^, Maarten de Bakker^1^, Mark W. Konijnenberg^1,2^, J. Frank W. Nijsen^1^

^1^ Radboud university medical center, Department of Medical Imaging, Nijmegen, The Netherlands

^2^ Erasmus university medical center, Department of Radiology and Nuclear Medicine, Rotterdam, The Netherlands

^*^ Corresponding author at [Lovisa.WestlundGotby@radboudumc.nl](mailto:Lovisa.WestlundGotby@radboudumc.nl)

^†^ These authors have contributed to the manuscript equally and share second authorship

## Calibration of Broad Quantification

To utilize Broad Quantification absolute quantification software, the Symbia Intevo Bold SPECT/CT scanner had been calibrated (sensitivity calibration and volume sensitivity calibration) according to the vendor instructions prior to the first measurement of this study. A holmium-166 (^166^Ho) point source of 201.18 MBq (volume 303 µL) was used for the sensitivity calibration (measured count rate was 4.5 kcts/s), and a uniform cylindrical phantom (inner diameter of 20 cm, an inner height of 20 cm, a volume of 6283 mL) with 0.041 MBq/mL was used for the volume sensitivity calibration. ^166^Ho-chloride was used for both the point source and the cylindrical phantom (HolmiumSolution, Quirem Medical B.V., Deventer, The Netherlands), additionally the ^166^Ho-chloride solution in the cylindrical phantom was saturated with 50 mM ethylenediaminetetraacetic acid. The ^166^Ho activities were measured in a dose calibrator (manufactured in 2019, equipped with ionization chamber VIK-202, Comecer, Joure, The Netherlands). The dead-time estimates (read from the workstation during the acquisition of the data, note that this is a snapshot of the displayed values on the . ‘Tomo Acquisition’-activity, ‘Analyzer’-tab) were approximately 6.75% for the point source and 3.25% cylindrical phantom. The resulting calibration factors for the Broad Quantification software were:

- *Sensitivity calibration detector 1* = 7.5 cps/MBq
- *Sensitivity calibration detector 2* = 7.6 cps/MBq
- *Volume Sensitivity Factor* = 1.22

## Counting rate performance

To evaluate the difference in counting rate performance (CRP) of the two projection datasets (uncorrected vs. TrueCalc corrected) generated from each acquisition of the Jaszczak phantom, the observed counting rate in the projection data of the photopeak window was plotted as a function of the total activity in the phantom at each measurement time point. In order to characterize the loss of counts that the system experiences as a result of deadtime effects, the paralyzable detector model (PDM) [1, 2] was fitted to the data using a nonlinear least squares method. This model is defined as $R_{obs}=\alpha Ae^{-A\tau}$, where $R_{obs}$ is the observed counting rate [kcps], $\alpha$ is a scaling factor to convert the activity to count rate [kcps/MBq], $A$ is the total activity in the phantom [MBq], and $\tau$ represents the system deadtime [μs]. Ideal CRP (no dead-time losses) is characterized by $R_{ideal}=\alpha A$. Based on the PDM we define the percent error (PE) [%] of the measured data compared to the ideal behavior as $\mathrm{PE}=100\times\left( \frac{R_{obs}-R_{ideal}}{R_{ideal}} \right)$.

The plot of the counting rate performance can be found in Supplemental figure 1. In this figure we can see that the uncorrected data features a slightly higher counting rate performance than the TrueCalc corrected data. The coefficient of determination ($R^{2}$) for the PDM fit was 0.9990 for both datasets. According to the PDM, the line describing a loss-less detector response (ideal CRP) is slightly steeper for the uncorrected data compared to the TrueCalc corrected data. The estimated losses (PEs) are however comparable for the two datasets. Due to dead-time effects and pulse pile-up, already the data for time point 13 (206-217 MBq) deviates from the straight lines of the ideal CRPs. For a scanner-specific conversion factor (CF_homogeneous_) based on a single time point (activity level) to be suitable to use over a range of measured activities, it is crucial that the detector response is linear in that region.


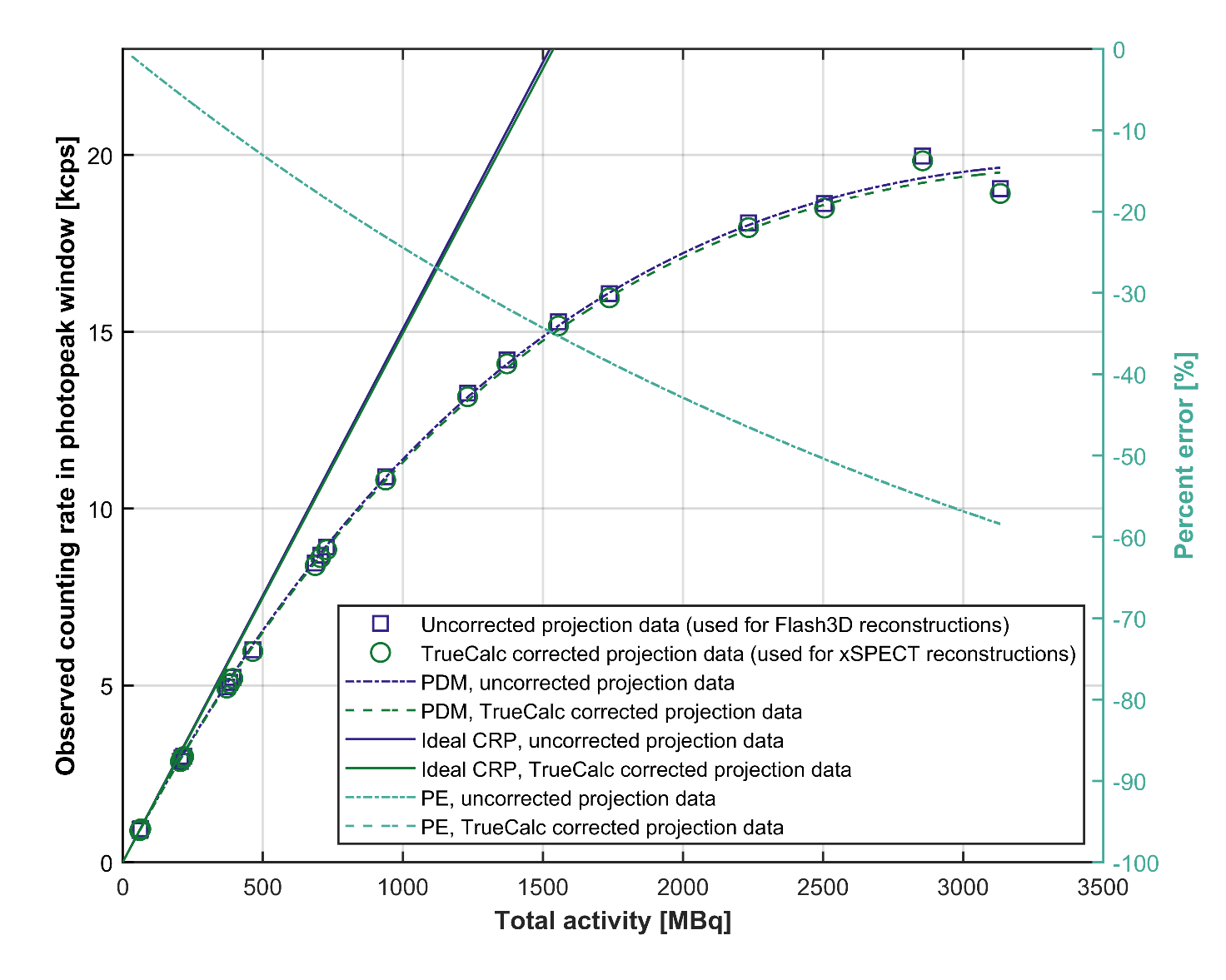


**Supplemental figure 1** Observed counting rate in the photopeak window of the two projection datasets (uncorrected and TrueCalc corrected) as a function of the activity in the Jaszczak phantom. The PDM has been fitted to the data (purple dash-dotted line and green dashed line), the corresponding ideal CRPs are depicted (purple and green solid lines) and the PEs (read out on the y-axis on the right hand side) has been calculated for the whole range of measured activities. The uncorrected data present a slightly higher counting rate than the TrueCalc corrected data, and therefore a slightly steeper line for the ideal CRP. The PEs are comparable for the two datasets

## Choice of image reconstruction parameters

The image reconstruction has been optimized based on a subset of the time points acquired for the Jaszczak phantom (time point 2, 4, 6, 9, 12). The aim of the optimization was to generate a high contrast recovery coefficient (CRC) and a high contrast-to-noise ratio (CNR) for the spherical inserts while at the same time limiting the coefficient of variation (COV; noise) in the background compartment. The image reconstruction parameters were based on a sweep of the number of updates needed to calculate the image. The number of updates were calculated as

$$\#updates=\#iterations \times\#subsets.$$

As previously have been shown for lutetium-177 [3], the subdivision between the number of iterations and the number of subsets has no impact on the noise build-up in Flash3D reconstructions, and therefore the number of subsets can be increased to accelerate the reconstruction. On the contrary, the xSPECT reconstructions should be performed using only 1 subset in order to minimize the noise build-up in the image (this is also in accordance with the manufacturer recommendations). The number of subsets were therefore fixed to 8 and 1 for Flash3D and xSPECT reconstructions, respectively.

The CRC was defined as

$CRC=\frac{\frac{\mu_{s}}{\mu_{BG}}-1}{S:BG_{\mathrm{ratio}}-1}$,

where $\mu_{s}$ is the mean counts per second (Flash3D reconstructions) or mean activity concentration [Bq/mL] (xSPECT reconstructions) in the spherical inserts, $\mu_{BG}$ is the mean counts per second (Flash3D reconstructions) or mean activity concentration [Bq/mL] (xSPECT reconstructions) in the background compartment of the Jaszczak phantom, and $S:BG_{\mathrm{ratio}}$ is the sphere-to-background activity concentration ratio (which was equal to 10 for this experiment). The CNR was defined as

$CNR=\frac{\mu_{s}-\mu_{BG}}{\sigma_{BG}}$ ,

where $\mu_{s}$ and $\mu_{BG}$ have the same definitions as above, and $\sigma_{BG}$ is the standard deviation of the counts per second (Flash3D reconstructions) or standard deviation of the activity concentration [Bq/mL] (xSPECT reconstructions) in the in the complete background compartment of the Jaszczak phantom, respectively. The COV was defined as

$COV=\frac{\sigma}{\mu}$ ,

where $\sigma$ is the standard deviation of the counts per second (Flash3D reconstructions) or of the activity concentration [Bq/mL] (xSPECT reconstructions) in a certain volume-of-interest (VOI), and $\mu$ is the mean counts per second (Flash3D reconstructions) or mean activity concentration [Bq/mL] (xSPECT reconstructions) (in the same VOI as the $\sigma$). Three different VOIs were considered for the COV analysis;

1. The complete background (6659.16 mL; *Background*),
2. A cylindrical VOI in the background compartment of the Jaszczak phantom, at the end were the lid is attached (positioned 30 mm off-center away from the largest sphere, 83.02 mL; *Homogeneous VOI top*),
3. A cylindrical VOI centered in-between the rods of the spherical inserts (197.13 mL; *Homogenous VOI bottom*).

All VOIs were semi-automatically created based on the known phantom geometry and CT based positioning on a high resolution CT (voxel size $0.98\times0.98\times1.5 \text{m}\text{m}^{\text{3}}$), and their corresponding volumes can be found in Supplemental table 1.

Supplemental table 1 Name and volumes of the volume-of-interests (VOIs) segmented for the image analysis (Jaszczak phantom). The volume of the VOIs differ from the true volumes because of the discretization of the CT voxel space and because the segmented sphere centers were forced to be positioned at the center of a voxel. The volumes were constant for all datasets/time points.

| **Name VOI** | **Segmented volume [mL]** |
| --- | --- |
| Sphere 1 | 0.44 |
| Sphere 2 | 1.91 |
| Sphere 3 | 4.16 |
| Sphere 4 | 8.06 |
| Sphere 5 | 15.27 |
| Sphere 6 | 114.92 |
| Background | 6659.16 |
| Homogeneous VOI top | 83.02 |
| Homogeneous VOI bottom | 197.13 |

The calculated CRC, CNR, and COV for Flash3D and xSPECT reconstructions can be found in Supplemental figure 1 and Supplemental figure 2, respectively. As mentioned before, we aim to maximize the CRC and CNR while minimizing the COV. In these figures we can see that these maxima and minima never occur at the same number of updates; many updates is generally more favorable for CRC maximization, few updates are generally more favorable for COV minimization, and a trade-off is favorable for CNR maximization. Additionally, we also see that the activity present in the phantom (different timepoints) has an impact on when the extremes appear. To be able to use the same reconstruction parameters across all the different timepoint we therefore chose

- Flash3D: 10 iterations and 8 subsets, no postprocessing filtering (to preserve quantitative accuracy),
- xSPECT: 36 iterations and 1 subset, 15 mm full width at half maximum Gaussian postprocessing filtering (as per manufacturer recommendations)

as these parameters balances these three outcome metrics (CRC, CNR, COV) for all timepoints.


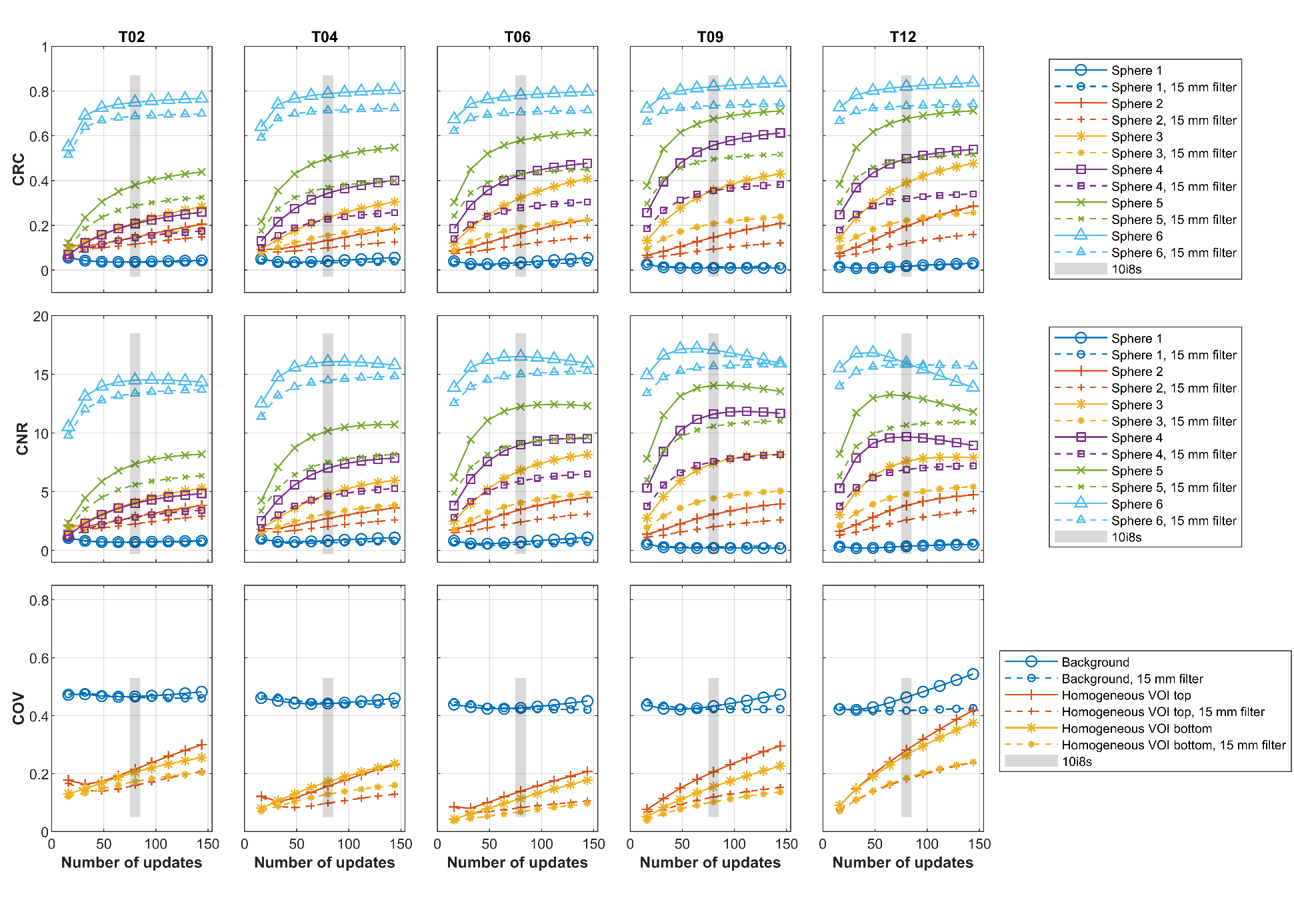


**Supplemental figure 2** CRC, CNR, and COV (rows) for five different time points (columns) of the Flash3D reconstructions. The x-axis of each subplot represents the number of updates in the image. The number of subsets was fixed to 8. The grey shaded area represents the chosen number of updated for the Flash3D image reconstruction (10 iterations and 8 subsets)


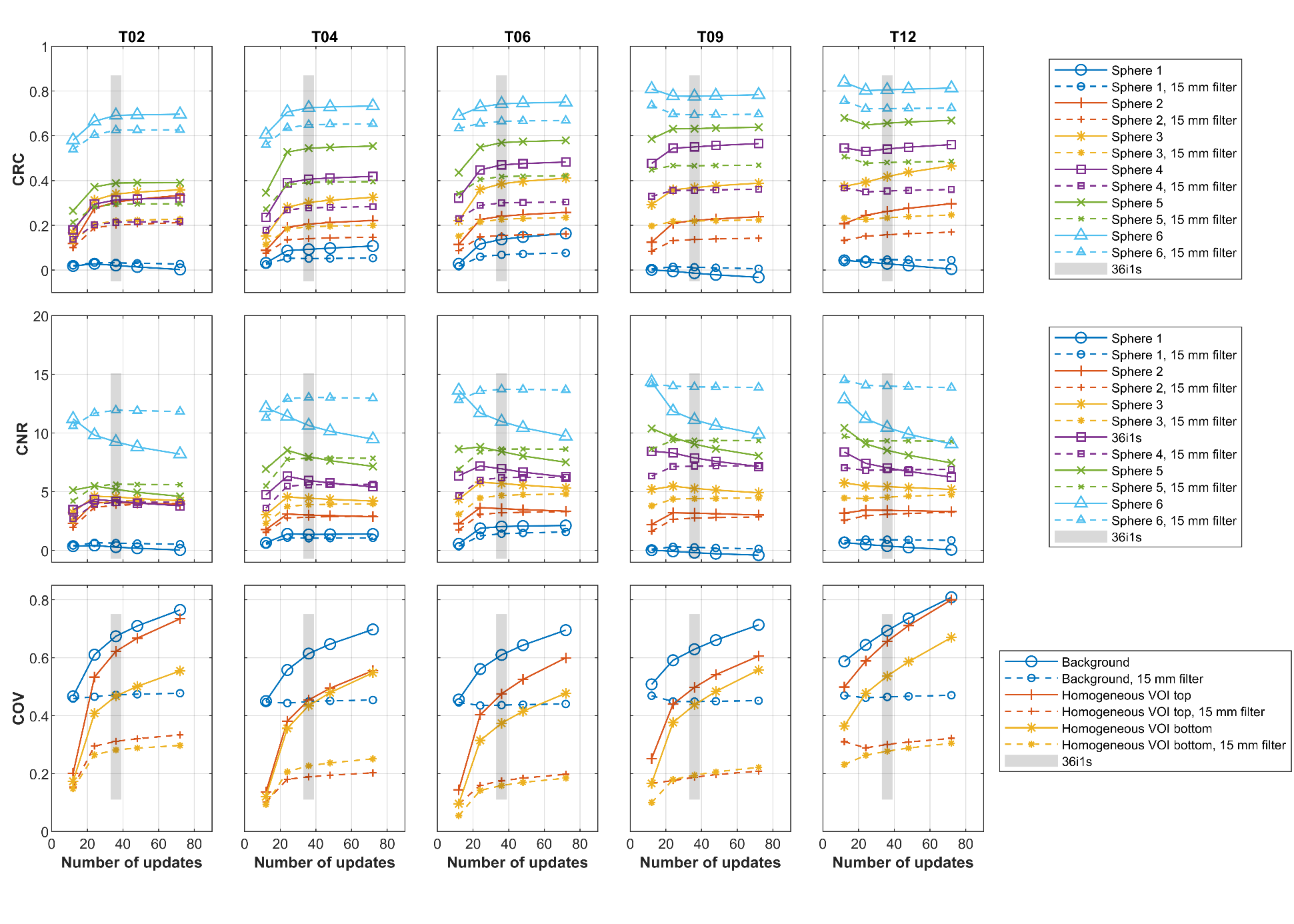


**Supplemental figure 3** CRC, CNR, and COV (rows) for five different time points (columns) of the xSPECT reconstructions. The x-axis of each subplot represents the number of updates in the image. The number of subsets was fixed to 1. The grey shaded area represents the chosen number of updated for the xSPECT image reconstruction (36 iterations and 1 subset)

## References

1. Sorenson JA (1975) Deadtime characteristics of anger cameras. J Nucl Med 16:284–288

2. Silosky M, Johnson V, Beasley C, Kappadath SC (2013) Characterization of the count rate performance of modern gamma cameras. Med Phys. https://doi.org/10.1118/1.4792297

3. Tran-Gia J, Lassmann M (2019) Characterization of Noise and Resolution for Quantitative 177 Lu SPECT/CT with xSPECT Quant. J Nucl Med 60:50–59
